# Q fever outbreak in the terraced vineyards of Lavaux, Switzerland

**DOI:** 10.1002/nmi2.37

**Published:** 2014-07-16

**Authors:** C Bellini, I Magouras, C Chapuis-Taillard, O Clerc, E Masserey, G Peduto, O Péter, S Schaerrer, G Schuepbach, G Greub

**Affiliations:** 1 Service of Infectious Diseases, Riviera Regional HospitalVevey, Switzerland; 2 Veterinary Public Health Institute, Vetsuisse faculty, University of BernBern, Switzerland; 3 Service of Infectious Diseases, Medical Centre of VidyLausanne, Switzerland; 4 Service of Infectious Diseases, Centre Hospitalier Universitaire Vaudois and University of LausanneLausanne, Switzerland; 5 Service of Public Health, Canton of VaudLausanne, Switzerland; 6 Service of Consumption and Veterinary Affairs, Canton of VaudLausanne, Switzerland; 7 Service of Infectious Diseases, Central Institute of ValaisSion, Switzerland; 8 Institute of Veterinary Bacteriology, Vetsuisse faculty, University of ZurichZurich, Switzerland; 9 Institute of Microbiology, Centre Hospitalier Universitaire Vaudois and University of LausanneLausanne, Switzerland

**Keywords:** *Coxiella burnetii*, environment, outbreak investigation, Q fever, sheep, veterinary investigation

## Abstract

*Coxiella burnetii* infection (Q fever) is a widespread zoonosis with low endemicity in Switzerland, therefore no mandatory public report was required. A cluster of initially ten human cases of acute Q fever infections characterized by prolonged fever, asthenia and mild hepatitis occurred in 2012 in the terraced vineyard of Lavaux. Epidemiological investigations based on patients' interviews and veterinary investigations included environmental sampling as well as *Coxiella*-specific serological assay and molecular examinations (real-time PCR in vaginal secretions) of suspected sheep. These investigations demonstrated that 43% of sheep carried the bacteria whereas 30% exhibited anti-*Coxiella* antibodies. Mitigation measures, including limiting human contacts with the flock, hygiene measures, flock vaccination and a public official alert, have permitted the detection of four additional human cases and the avoidance of a much larger outbreak. Since November 2012, mandatory reporting of Q fever to Swiss public health authorities has been reintroduced. A close follow up of human cases will be necessary to identify chronic Q fever.

## Introduction

Q fever is an infection caused by *Coxiella burnetii*, a naturally intracellular Gram-negative bacterium. ‘Q’ stands for Query and this name was first used in 1935 during an outbreak of febrile illness among abattoir workers in Brisbane, Queensland (Australia) when the causative agent was still unknown 1,2.

Common reservoirs of this worldwide zoonotic disease are wild and domestic animals, especially sheep, goats, cattle and occasionally pets. Infected animals are often asymptomatic, but abortions and other reproductive disorders can manifest. Shedding occurs in urine, milk, faeces, and in particular through birth products from infected animals 2–4.

As spore-like forms of *C. burnetii* can survive for months in the environment and the bacteria have been documented in dust samples 5,6, infections do not necessarily require direct contact with diseased animals. Inhalation of aerosolized particles is the primary transmission route 7,8. Infections through direct skin contact, ingestion of contaminated raw milk and goat cheese have also been described 2. Rare human-to-human transmissions following contact with infected placenta, human milk exposure and via blood transfusions have also been documented 2,9–11.

After an incubation period of around 20 days (range 9–39 days), non-immune exposed persons develop a primary infection. Acute infection remains asymptomatic in around 60% of cases 3,12–14. For the remaining 40% of cases, acute Q fever usually manifests as self-limited, flu-like illness, interstitial pneumonia or acute hepatitis 15. Spontaneous abortion or premature delivery can occur in pregnant women 15,16. Only 5% of the symptomatic persons will require hospitalization 13.

In 1–5% of all infections, Q fever progresses into a chronic form, whose localization (e.g. endocarditis, vascular infection, granulomatous hepatitis, osteomyelitis) depends on host risk factors and *C. burnetii* isolate groups 3,15,17,18.

Recently the Netherlands experienced the largest outbreak ever recorded, with more than 4000 human cases by 2011 19,20. In Switzerland, a European country of about 8 000 000 inhabitants, the incidence of Q fever is about 0.15 cases per 100 000 inhabitants, corresponding to around 10–12 infections per year (http://www.bag.admin.ch/dokumentation/publikationen). Given this low incidence, reports of human cases to public health authorities were no longer mandatory after 1999, so its epidemiology is now largely unknown (http://www.bag.admin.ch/dokumentation/publikationen). Outbreaks were rarely reported, and only one large Swiss outbreak has been documented in the ‘Val de Bagnes’ in 1983 21.

In Switzerland, abortions of cloven-hoofed animals have to be reported to the veterinary authorities. Between 2002 and 2011, 583 abortions due to *C. burnetii* infections were reported, 82% of them in cattle, 12% in goats and 6% in sheep (http://www.bvet.admin.ch/themen/03605/index.html?lang=fr). In the last 5 years, around 60–80 animal infections occurred each year (http://www.bvet.admin.ch/themen/03605/index.html?lang=fr). Screening of the milk products from Swiss animals has shown that *C. burnetii* contamination occurred in <5% of cattle products, and never in sheep or goat samples 22.

Because of the asymptomatic nature of most human infections and the rarity of the disease in Switzerland, Q fever is poorly known among general practitioners, and only rarely considered in the differential diagnosis of flu-like illnesses. We report the results of an outbreak investigation of 14 cases in the terraced vineyards of Lavaux, Canton of Vaud, Switzerland, and describe related environmental and veterinary investigations.

## Methods

### Epidemiological description

Between February and May 2012 an unusually high number of hospitalized cases was observed in three different Swiss hospitals, located in Vevey (*n* = 1) and in Lausanne (*n* = 2). The initial cluster of ten cases was reported to the public health authorities. Considering the rarity of this disease, and the lack of knowledge about this infection among general practitioners, we suspected that these few cases indicated a much larger outbreak and a larger investigation was initiated allowing the identification of 4 additional cases. The patients presented acute and prolonged febrile illness up to 2 weeks associated with asthenia and hepatitis. Table1 summarizes the clinical presentation of the 14 cases and Table2 shows the rate of the majority of symptoms and signs documented for these 14 cases. These cases included two episodes of biopsy-proven granulomatous hepatitis (patients 1 and 3) and one vertebral osteomyelitis (patient 6). Acute Q fever diagnoses were based on positive serologies, defined as a phase II IgM titre ≥40 IU/L and phase II IgG titre ≥40 IU/L (immunofluorescence assay) 13. Briefly, sera were screened by indirect immunofluorescence assay at a starting dilution of 1/20 using *Coxiella burnetii* phase I and II antigens (strain Nine Miles, kindly provided by Dr W. Burgdorfer, Rocky Mountain Laboratories, Hamilton, USA) 23. Fluorescein isothiocyanate goat anti-human specific IgG and IgM conjugates (BioMérieux, Marcy-l'Etoile, France) were used for detection. Serology was often performed after more than 10 days of intra-hospital investigations, secondary to infectious disease consultation. In seven cases (patients 5,7,8,11,12,13,14), for which initial serology was negative, seroconversion was documented 14 days later. Only in one case (patient 12), could *C. burnetii* DNA be detected by real-time PCR in serum 24. The PCR was also positive in one of the two liver biopsies that were performed (patient 1) and in another case in a bone biopsy (patient 6). Two cases had predisposing risk factors for chronic Q fever (patients 1 and 3).

**Table 1:** Patient descriptions, including predisposing and exposure risk factors, clinical presentation and evolution

No.	Age (years) at diagnosis; gender	First symptoms (month/year)	Clinical presentation	Diagnosis of *Coxiella burnetii* infection	Predisposing conditions	Anamnestic exposure risk factors	Treatment and evolution including clinical investigations
1	62; M	04/2011^a^	Flu-like symptoms Granulomatous hepatitis	Positive serology and PCR in liver biopsy (granuloma)	TNF-α inhibitors for Bechterew disease	Regular visits to friends in the farm	Doxycycline 100 mg twice daily for 21 days normal at screening and at 6 months; TA-CT normal at 1 year Recovery
2	66; M	06/2011^b^	Flu-like symptoms Hepatitis	Positive serology	None	Live in the region; eat unpasteurized goat cheese and local market vegetables	Doxycycline 100 mg twice daily for 14 days normal at screening and at 4 months; TEE normal at 4 months; bone scintigraphy normal at 4 months Recovery after 1 year
3	59; M	02/2012^b^	Flu-like symptoms Granulomatous hepatitis	Positive serology Negative PCR in liver biopsy	Aortic bicuspidia and aneurysm	Regular visits to friends in the farm	Doxycycline plus hydroxylchloroquine (skin rash); switch to doxycycline plus rifampin TTE showing aortic bicuspidia and aneurysm of ascending aorta at screening Resolution of hepatitis
4	64; F	04/2012^b^	Flu-like symptoms Hepatitis	Positive serology	None	Live in the region; eat local market vegetables	Doxycycline 100 mg twice daily for 14 days TTE normal at screening Recovery after 3 months
5	44; M	04/2012^b^	Flu-like symptoms Hepatitis	Documented seroconversion	None	Live in the region; eat local market vegetables	Doxycycline 100 mg twice daily for 14 days TTE normal at screening Recovery after 3 months
6	57; M	04/2012	Spondylodiscitis Hepatitis	Positive serology and PCR on vertebral biopsy	Vertebral trauma 2 months earlier	Regular visits to friends in the farm; eat local market vegetables	Doxycycline plus hydroxylchloroquine (cutaneous lupus) switch to ciprofloxacin plus rifampin TEE and TA-CT normal at screening Resolution of hepatitis Persistence of vertebral inflammation on vertebral MRI at 3 months
7	44; M	05/2012	Flu-like symptoms Hepatitis	Documented seroconversion	None	Work in the region	Doxycycline 100 mg twice daily for 14 days TTE normal at screening Recovery after 14 days
8	73; F	05/2012	Flu-like symptoms Hepatitis Interstitial pneumonia	Documented seroconversion Negative PCR in lung secretions	Chronic renal impairment; sequels following breast radiotherapy for cancer	Regular walk in the region; eat local market vegetables	Doxycycline 100 mg twice daily for 14 days Recovery after 3 months
9	44; F	05/2012	Flu-like symptoms	Positive serology	None	Live in the farm; have direct contact with goats; eat farm vegetables	Doxycycline 100 mg twice daily for 14 days TEE normal at screening Recurrence of symptoms after 3 months and new elevation of phase 2 antibodies; TA-CT normal Recovery after 14 days of ciprofloxacine treatment
10	51; M	05/2012	Flu-like symptoms	Positive serology	None	Live in the farm; have direct contact with goats; eat farm vegetables	No treatment Recovery
11	48; M	07/2012	Flu-like symptoms Mild hepatitis	Documented seroconversion	None	Live in the region; eat unpasteurized goat cheese and local market vegetables	No treatment Recovery
12	65; M	08/2012	Flu-like symptoms Mild hepatitis	Documented seroconversion Positive PCR in first serum while negative serology	None	Live in the region	No treatment TEE normal at screening Recovery
13	48; M	07/2012	Flu-like symptoms Granulomatous hepatitis	Documented seroconversion	None	Live in the region	Doxycycline 100 mg twice daily for 10 days TEE normal at screening Recovery
14	40; F	08/2012	Flu-like symptoms Hepatitis	Documented seroconversion	Pregnancy	Live in the region	Doxycycline 100 mg twice daily for 21 days TEE normal Recovery

MRI, magnetic resonance imaging; TA-CT, thoracoabdominal computer tomography; TEE, transoesophageal echocardiography; TNF, tumour necrosis factors; TTE, transthoracic echocardiography.

a Following epidemiological investigations, discovery of patient 1 (probably the first case), who used to regularly meet friends in the index farm.

b Announcement to public health authorities was made when cases 1 to 5 had been identified.

**Table 2:** Frequency of some clinical features at the moment of diagnosis among the 14 human cases documented during the outbreak

Symptoms	Frequency	% in this series of 14 cases
Persistent fever (>14 days)	14	100
Fatigue	12	86
Liver enzyme elevation	12	86
Profuse night sweats	10	71
Inappetence	10	71
Severe headache	8	57
Myalgia	8	57
Diffuse arthralgia	6	43
Nausea	6	43
Mild weight loss	3	21
Diarrhoea	3	21
Cough	2	14
Subjective palpitations	1	7

### Epidemiological investigation and public health measures

Investigations initially included patient interviews, review of medical records and evaluation of risk factors for exposure to Q fever (working and living places, environmental exposure, animal contact and food habits), which identified a sheep farm as a possible source of the outbreak (coined hereafter as the ‘index farm’).

Based on this information, a veterinary investigation was launched in June 2012. At that time, all the sheep had already been transferred to Alpine pastures near the lake of l'Hongrin, in the Canton of Vaud. On the farm, most manure had been removed and the floor had been flushed with water. As a first step, on-farm sampling of dust and manure as well as an interview with the farmer were carried out. In total, five dust samples were taken from horizontal surfaces such as feed troughs (*n* = 2) and windowsills (*n* = 3), as described elsewhere 25. Furthermore, six manure samples were collected in sterile vials from different locations on the ground. All samples were frozen at −20°C until further processing. In July 2012, 52 sheep, part of the flock suspected as the source of the outbreak and now located in the Alps, were randomly sampled for serology and real-time PCR examinations of vaginal swabs. Sera were tested for the presence of antibodies against *C. burnetii* using a commercial ELISA test (CHEKIT Q fever Antibody ELISA Test Kit, IDEXX, Liebefeld, Switzerland). For dust samples and vaginal swab examinations, DNA was extracted using a QIAamp® cador® Pathogen Kit (Qiagen, Hilden, Germany) and for manure samples with QIAamp® DNA Stool Mini Kit (Qiagen), as described by the manufacturer. Real-time PCR was performed using a commercial assay (LSI Taqvet *C. burnetii*, Laboratoire Service International, Lissieu, France) according to the manufacturer's instructions.

Meanwhile, local physicians were informed about the ongoing outbreak by an official communication. Furthermore, the outbreak was brought to the attention of the public through local newspapers, television and other media. Voluntary reporting of every new case was introduced. Based on the literature review, particularly the experience gained from the outbreak in the Netherlands, screening of all blood donors was performed using a *Coxiella*-specific Taqman real-time PCR 24, to limit the risk of human-to-human transmission by blood transfusion 26.

In animals, follow-up vaginal swabs (*n* = 50) were taken for PCR examinations 1 month after the initial sampling (August 2012).These samples also included sheep that were in contact with animals from the outbreak farm while on the Alps, but belonging to three different owners (animal sample sizes five, five and ten, respectively). Furthermore, two additional dust samplings (dust sample sizes ten and five, respectively) were carried out 1 month apart (July and August 2012) following cleaning and disinfection of the index farm in Lavaux.

## Results

All except two patients (patients 9 and 10) lived in urban areas and did not report direct contact with animals, except for occasional recreational walks through the rural area of Lavaux. All except two patients did not report ingestion of unpasteurized milk products. Consumption of local market products originating from the rural area of Lavaux was common, although the precise origin of these could not be determined. Based on the predisposing and exposure risk factors identified through anamnesis (Table1), we considered that transmission occurred by inhalation of contaminated aerosols, although ingestion of contaminated vegetables could not be completely excluded.

Three infected patients (patients 1, 3 and 6) regularly visited friends on the suspected farm, where four members of the farmer's family showed symptoms of self-limited febrile illness. Serology confirmed an acute infection with *C. burnetii* in these patients (patients 9 and 10) whereas no serology was performed for their two children. Taken together, these data imply that the Q fever outbreak originated from this farm.

This index farm housed approximately 400 meat-type sheep (ewes with their lambs) and is located in the northern part of Lavaux. The farmer owned an additional 750 sheep, which were kept at different locations in the canton of Vaud. All sheep were brought to the Alps at the end of May and grazed together with sheep belonging to another 11 farmers, making a total number of approximately 2000 animals. According to the farmer, only two abortions were observed at the index farm following shearing. All environmental samples taken at the farm tested positive for the presence of *C. burnetii* DNA. Five out of 11 environmental samples (one dust and four manure samples) were strong positives, whereas the remaining six samples (four dust and two manure samples) tested as moderate positives.

In agreement with the farmer, veterinarians went to the Alps and randomly sampled 52 sheep for serological and real-time PCR examinations. Since the sheep on all locations (i.e. farms) were grouped together, it was not possible to specifically sample only those sheep originating from the index farm. Nevertheless, identification of animals was possible at ‘ownership’ level. Fifteen out of 50 tested sheep (30%) tested positive for the presence of *C. burnetii* antibodies. Two serum samples with questionable results were interpreted as negative, whereas two samples could not be tested because of insufficient amounts of blood. Real-time PCR examination of vaginal swabs revealed 43% (22/51) of the samples to be positive for *C. burnetii* DNA. For one swab no result was obtained due to inhibition of the PCR. As for the environmental samples, vaginal swab PCR results were interpreted as weak, moderate and strong positives (qualitative result). Table3 shows the relationship between serological status and vaginal shedding of 49 sheep for which both data are available. As expected, not all seropositive animals were shedding *Coxiella* whereas not all shedders (i.e. PCR-positive animals) were already seropositive at the time of investigation.

**Table 3:** Comparison of molecular analysis for *Coxiella burnetii*DNA from vaginal secretions and serological results (ELISA) of 49 tested sheep, for which paired results are available

	Positive ELISA (*n* = 15)	Negative ELISA (*n* = 34)	Total (*n* = 49)
Positive RT-PCR (*n* = 21)	7 (14%)	14 (29%)	21 (43%)
Negative RT-PCR (*n* = 28)	8 (16%)	20 (41%)	28 (57%)
Total (*n* = 49)	15 (31%)	34 (69%)	49 (100%)

The second round of PCR examinations, which also included sheep belonging to three additional farmers, gave 42 positive results out of 50 vaginal swabs tested. Positive samples were found in animals owned by all four farmers. Finally, two PCR-positive swabs were detected on each of the two subsequent dust samplings carried out 1 month apart.

In order to avoid additional human cases, the veterinary authorities ordered an extensive vaccination 27 of the infected sheep flock, combined with restrictions for all sheep, and hygiene measures on the index farm.

Following this alert, four further human cases were reported to local authorities (patients 11 to 14) and after these four cases, no additional cases were reported. A total of 1345 blood donors were tested negative for *C. burnetii* DNA by real-time PCR in blood 24.

## Discussion

This report describes an outbreak of Q fever with 14 human cases of severe infections that occurred in the vineyard of Lavaux, Switzerland. Diagnosis was mainly based on serology. One case presented osteomyelitis, a rare manifestation of Q fever, confirmed by real-time PCR. Another patient with pre-existing valvular anomalies and a vascular aneurysm was treated for a prolonged period for a suspected chronic Q fever. Because of the high number and severity of cases, public health measures were needed to mitigate the outbreak. Precise interviews of patients about their behaviour and risk factors were essential to identifying the possible source. Veterinary investigations permitted the identification of a possible reservoir, a flock of nearly 400 sheep in the northern region of Lavaux.

A number of human Q fever outbreaks linked to sheep farms near residential areas have already been described in many countries 28–31. Furthermore, 24 out of 40 outbreaks recorded between 1947 and 1999 in Germany were associated with sheep 32. In Switzerland, the last reported large Q fever outbreak in 1983 was linked to sheep descending from the Alps, causing 415 acute Q fever cases in humans residing along the route 21.

According to the farmer, only two abortions were observed on the index farm and these can be attributed to stress associated with shearing practices. However, in small ruminants shedding is not always linked to abortions and bacteria can also be shed following normal parturition 33,34. An association of human Q fever disease with visits to sheep farms housing newborn lambs and without history of excess abortions has already been described in the Netherlands 35. Interestingly, even a single lambing ewe has induced an outbreak affecting hundreds of people in Germany 36. In the present cluster of cases, the index farm housed approximately 200 ewes and their lambs born from winter to spring 2012. Human outbreaks of Q fever in Europe show a seasonal pattern with peaks occurring in spring and early summer 3,37 as was the case for this small outbreak.

In addition to birth products, infected animals also shed bacteria with urine, milk and faeces 2–4. In sheep, vaginal discharge and faeces are the most common shedding routes 38 and contribute to environmental contamination 5,6 and to infectious aerosol spread. We have examined the presence of *C. burnetii* in dust samples on surfaces inside farm housings as well as in manure remnants on the floor. The majority of strong positive PCR results were obtained from manure samples. Considering that the farmer had flushed the floor with water before sampling, these results support a massive contamination of manure with *C. burnetii*. The use of sheep manure as a fertilizer has indeed been suggested as a possible cause of Q fever infections in humans 39. In addition, during the 2007–2008 outbreaks in the Netherlands, a peak incidence of human cases has been associated with manure spreading 40.

As all sheep were grouped together when in the Alps, it was not possible to only sample those originating from the index farm (*n* = 400). However, movements of animals and equipment between the farms of the same owner are to be expected, hence all animals belonging to the owner of the index farm were treated as an epidemiological unit (*n* = 1150). PCR-positive vaginal samples were also detected in sheep belonging to three other farmers. Considering that farmers usually bring their animals to the same pastures every year, the origin of the infection cannot be proven based on these results because it could have been introduced and spread between the flocks already in previous years.

A positive serology only indicates past exposure and is not proof of active infection and bacterial shedding in the environment. The detection of shedding is of great public health significance and should always be included in Q fever outbreak investigations. In this outbreak, laboratory examinations revealed a large number of seropositive sheep (30%) and an even larger number of animals shedding *C. burnetii* in vaginal mucus (43%). Interestingly, only seven seropositive animals were also shedding at the same time. This is in accordance with previous data showing that seronegative ruminants might shed bacteria whereas seropositive animals do not necessarily shed 33,41. The animals were tested approximately 2 and 3 months following transport to the Alps, when lambing at the farms of origin was already completed. The positive PCR results from vaginal swabs support previous studies showing that sheep shed for longer in vaginal discharges than other ruminants following birthing 37.

To protect the population, all sheep were vaccinated, hygiene measures were applied at the index farm and movement restrictions were imposed to all sheep on the Alps. The persistence of PCR-positive dust samples following disinfection is attributed to the old premises of the farm that could not be effectively cleaned and disinfected. Testing all blood donors did not reveal any positive test, and we do not advise repeating this measure as routine, although in a large Q fever outbreak, such PCR testing could avoid human-to-human transmission by transfusion.

This cluster of cases permitted the improvement of both public and physician awareness of Q fever and helped consideration of this illness as a prolonged febrile illness. Indeed, its recognition is important for timely diagnosis and to avoid long-term consequences. Furthermore, this outbreak highlights the risk of Q fever transmission from animals to humans in residential areas located near farms. Considering the ongoing urbanization, strong cooperation of public health and infectious diseases specialists and veterinary scientists is crucial for the prevention and control of future Q fever outbreaks.

## References

[b1] Derrick EH (1937). “Q” fever a new fever entity: clinical features, diagnosis, and laboratory investigation. Med J Aust.

[b2] Raoult D, Marrie T, Mandell GL, Bennett JE, Dolin R (2010). *Coxiella burnetii* (Q fever). Principles and practice of infectious diseases.

[b3] Maurin M, Raoult D (1999). Q fever. Clin Microbiol Rev.

[b4] Baud D, Peter O, Langel C, Regan L, Greub G (2009). Seroprevalence of *Coxiella burnetii* and *Brucella abortus* among pregnant women. Clin Microbiol Infect.

[b5] Ratmanov P, Bassene H, Fenollar F (2013). The correlation of Q fever and *Coxiella burnetii* DNA in household environments in rural Senegal. Vector Borne Zoonotic Dis.

[b6] Kersh GJ, Wolfe TM, Fitzpatrick KA (2010). Presence of *Coxiella burnetii* DNA in the environment of the United States, 2006 to 2008. Appl Environ Microbiol.

[b7] Raoult D, Marrie T, Mege J (2005). Natural history and pathophysiology of Q fever. Lancet Infect Dis.

[b8] Woldehiwet Z (2004). Q fever (coxiellosis): epidemiology and pathogenesis. Res Vet Sci.

[b9] Report CDW (1977). Comment on Q fever transmitted by blood transfusion—United States (Editorial). Can Dis Wkly Rep.

[b10] Raoult D, Marrie T (1995). Q fever. Clin Infect Dis.

[b11] Carcopino X, Raoult D, Bretelle F, Boubli L, Stein A (2009). Q fever during pregnancy: a cause of poor fetal and maternal outcome. Ann N Y Acad Sci.

[b12] Fournier PE, Raoult D (2003). Comparison of PCR and serology assays for early diagnosis of acute Q fever. J Clin Microbiol.

[b13] Million M, Lepidi H, Raoult D (2009). Q fever: current diagnosis and treatment options. Med Mal Infect.

[b14] Parker NR, Barralet JH, Bell AM (2006). Q fever. Lancet.

[b15] Raoult D (2012). Chronic Q fever: expert opinion versus literature analysis and consensus. J Infect.

[b16] Baud D, Greub G (2011). Intracellular bacteria and adverse pregnancy outcomes. Clin Microbiol Infect.

[b17] Beare PA, Samuel JE, Howe D, Virtaneva K, Porcella SF, Heinzen RA (2006). Genetic diversity of the Q fever agent, *Coxiella burnetii*, assessed by microarray-based whole-genome comparisons. J Bacteriol.

[b18] Russell-Lodrigue KE, Andoh M, Poels MW (2009). *Coxiella burnetii* isolates cause genogroup-specific virulence in mouse and guinea pig models of acute Q fever. Infect Immun.

[b19] Wegdam-Blans MC, Wielders CC, Meekelenkamp J (2012). Evaluation of commonly used serological tests for detection of *Coxiella burnetii* antibodies in well-defined acute and follow-up sera. Clin Vaccine Immunol.

[b20] van Loenhout JA, Paget WJ, Vercoulen JH, Wijkmans CJ, Hautvast JL, van der Velden K (2012). Assessing the long-term health impact of Q-fever in the Netherlands: a prospective cohort study started in 2007 on the largest documented Q-fever outbreak to date. BMC Infect Dis.

[b21] Dupuis G, Petite J, Peter O, Vouilloz M (1987). An important outbreak of human Q fever in a Swiss Alpine valley. Int J Epidemiol.

[b22] Fretz R, Schaeren W, Tanner M, Baumgartner A (2007). Screening of various foodstuffs for occurrence of *Coxiella burnetii* in Switzerland. Int J Food Microbiol.

[b23] Peacock MG, Philip RN, Williams JC, Faulkner RS (1983). Serological evaluation of Q fever in humans: enhanced phase I titers of immunoglobulins G and A are diagnostic for Q fever endocarditis. Infect Immun.

[b24] Jaton K, Peter O, Raoult D, Tissot JD, Greub G (2013). Development of a high throughput PCR to detect *Coxiella burnetii* and its application in a diagnostic laboratory over a 7-year period. New Microbe New Infect.

[b25] de Bruin A, van der Plaats RQ, de Heer L (2012). Detection of *Coxiella burnetii* DNA on small-ruminant farms during a Q fever outbreak in the Netherlands. Appl Environ Microbiol.

[b26] Hogema BM, Slot E, Molier M (2012). *Coxiella burnetii* infection among blood donors during the 2009 Q-fever outbreak in the Netherlands. Transfusion.

[b27] Astobiza I, Barandika JF, Juste RA, Hurtado A, Garcia-Perez AL (2012). Evaluation of the efficacy of oxytetracycline treatment followed by vaccination against Q fever in a highly infected sheep flock. Vet J.

[b28] Gilsdorf A, Kroh C, Grimm S, Jensen E, Wagner-Wiening C, Alpers K (2008). Large Q fever outbreak due to sheep farming near residential areas, Germany, 2005. Epidemiol Infect.

[b29] Lyytikainen O, Ziese T, Schwartlander B (1998). An outbreak of sheep-associated Q fever in a rural community in Germany. Eur J Epidemiol.

[b30] Wallensten A, Moore P, Webster H (2010). Q fever outbreak in Cheltenham, United Kingdom, in 2007 and the use of dispersion modelling to investigate the possibility of airborne spread. Euro Surveill.

[b31] Panaiotov S, Ciccozzi M, Brankova N (2009). An outbreak of Q fever in Bulgaria. Ann Ist Super Sanita.

[b32] Hellenbrand W, Breuer T, Petersen L (2001). Changing epidemiology of Q fever in Germany, 1947–1999. Emerg Infect Dis.

[b33] Berri M, Souriau A, Crosby M, Crochet D, Lechopier P, Rodolakis A (2001). Relationships between the shedding of *Coxiella burnetii,* clinical signs and serological responses of 34 sheep. Vet Rec.

[b34] Roest HJ, van Gelderen B, Dinkla A (2012). Q fever in pregnant goats: pathogenesis and excretion of *Coxiella burnetii*. PLoS One.

[b35] Whelan J, Schimmer B, de Bruin A, van Beest Holle MR, van der Hoek W, ter Schegget R (2012). Visits on ‘lamb-viewing days’ at a sheep farm open to the public was a risk factor for Q fever in 2009. Epidemiol Infect.

[b36] Porten K, Rissland J, Tigges A (2006). A super-spreading ewe infects hundreds with Q fever at a farmers' market in Germany. BMC Infect Dis.

[b37] Arricau-Bouvery N, Rodolakis A (2005). Is Q fever an emerging or re-emerging zoonosis?. Vet Res.

[b38] Rodolakis A, Berri M, Hechard C (2007). Comparison of *Coxiella burnetii* shedding in milk of dairy bovine, caprine, and ovine herds. J Dairy Sci.

[b39] Berri M, Rousset E, Champion JL (2003). Ovine manure used a a garden fertiliser as a suspected source of human Q fever. Vet Rec.

[b40] Delsing CE, Kullberg BJ (2008). Q fever in the Netherlands: a concise overview and implications of the largest ongoing outbreak. Neth J Med.

[b41] Arricau Bouvery N, Souriau A, Lechopier P, Rodolakis A (2003). Experimental *Coxiella burnetii* infection in pregnant goats: excretion routes. Vet Res.

